# Supplementary material for: A Comparative Analysis of the Wound Healing-Related Heterogeneity of Adipose-Derived Stem Cells Donors
Source: Pharmaceutics. 2022 Oct 6;14(10):2126. doi: 10.3390/pharmaceutics14102126 (PMC9608503; doi:10.3390/pharmaceutics14102126)
Supplement: Supplementary file 1 [file pharmaceutics-14-02126-s001.zip › pharmaceutics-1916796-supplementary.pdf]

pharmaceutics

MDPI

# Supplementary Materials: A Comparative Analysis of the Wound Healing-related Heterogeneity of Adipose-derived Stem Cells Donors

**Guoqiang Ren, Qiuyue Peng, Jeppe Emmersen, Vladimir Zachar, Trine Fink and Simone R. Porsborg**

**Table S1.** The up-regulated gene information.

| | FPKM | | | ASC-105 / ASC-101 | | ASC-106 / ASC-101 | |
|---|---|---|---|---|---|---|---|
| Gene Symbol | ASC-101 | ASC-105 | ASC-106 | log2 | FDR | log2 | FDR |
| LIMS4 | 1.43 | 3.39 | 3.26 | 1.25 | 0.000559 | 1.19 | 0.000991 |
| POC1B-GALNT4 | 0 | 0.11 | 0.42 | 3.46 | 0.00556 | 5.39 | 0.00000000002 |
| CORO7-PAM16 | 0 | 0.13 | 0.17 | 3.70 | 0.02 | 4.09 | 0.00311 |
| FSBP | 0.29 | 0.99 | 0.80 | 1.77 | 0.00000001 | 1.46 | 0.0000173 |
| U2AF1L5 | 3.44 | 10.03 | 8.59 | 1.54 | 0.000000000037 | 1.32 | 0.00000003 |
| LOC102724770* | 0 | 2.23 | 1.48 | 7.80 | 0.000000000024 | 7.21 | 0.0000001 |
| EFS* | 2.69 | 11.17 | 7.90 | 2.05 | 4.1E-56 | 1.55 | 5.4E-26 |
| SPON1*# | 6.03 | 19.22 | 18.12 | 1.67 | 2.1E-140 | 1.59 | 5.64E-119 |
| OLFM1 | 1.39 | 2.79 | 4.13 | 1.01 | 0.00478 | 1.57 | 0.000000001 |
| LINC01638 | 2.69 | 6.39 | 8.79 | 1.25 | 0.00119 | 1.71 | 0.0000006 |
| SLC12A7 | 0.61 | 2.25 | 1.85 | 1.88 | 1.9E-20 | 1.60 | 0.00000000000031 |
| STAG3 | 0.22 | 0.58 | 0.46 | 1.40 | 0.00217 | 1.06 | 0.03 |
| LOC107986352 | 0 | 0.13 | 0.23 | 3.70 | 0.01 | 4.52 | 0.000122 |
| CORO1A | 0.21 | 0.71 | 1.01 | 1.76 | 0.03 | 2.27 | 0.000223 |
| LZTS1* | 0.50 | 2.25 | 1.25 | 2.17 | 2.9E-26 | 1.32 | 0.0000002 |
| GALNT5 | 3.56 | 7.62 | 8.41 | 1.10 | 2,00E-23 | 1.24 | 1.4E-36 |
| MGAT4A | 0.02 | 0.14 | 0.09 | 2.81 | 0.01 | 2.17 | 0.02 |
| CHRNA5 | 0.08 | 0.30 | 0.36 | 1.91 | 0.02 | 2.17 | 0.0041 |
| GBP5 | 0.08 | 0.39 | 0.33 | 2.29 | 0.000566 | 2.04 | 0.0041 |
| HSPA12B | 0.10 | 0.37 | 0.44 | 1.89 | 0.01 | 2.14 | 0.0041 |
| WDR17 | 0.12 | 0.27 | 0.40 | 1.17 | 0.00000621 | 1.74 | 0.05 |
| COL4A4* | 0.04 | 0.31 | 1.46 | 2.95 | 9.9E-57 | 5.19 | 0.00000000088 |
| COL10A1* | 0.62 | 3.97 | 4.34 | 2.68 | 1.1E-42 | 2.81 | 4.2E-38 |
| COL11A1*# | 6.65 | 17.44 | 14.44 | 1.39 | 1.2E-72 | 1.12 | 1.43E-130 |
| HAPLN1* | 0 | 0.60 | 0.17 | 5.91 | 0.000451 | 4.09 | 0.000000000000061 |
| TMEM37 | 0.04 | 0.54 | 0.35 | 3.75 | 0.03 | 3.13 | 0.0019 |
| KRT80 | 0.08 | 0.32 | 0.33 | 2 | 0.0041 | 2.04 | 0.00568 |
| PIK3R6* | 0.08 | 0.46 | 0.71 | 2.52 | 0.0000007 | 3.15 | 0.000838 |
| CDC42EP5# | 6.31 | 21.57 | 19.81 | 1.77 | 4.8E-21 | 1.65 | 1.3E-26 |
| TEKT4 | 0.17 | 0.88 | 0.58 | 2.37 | 0.02 | 1.77 | 0.00839 |
| GPAT2 | 1.59 | 3.38 | 3.26 | 1.09 | 0.0000005 | 1.04 | 0.0000001 |
| FGD5 | 0.33 | 1.11 | 0.67 | 1.75 | 0.0024 | 1.02 | 0.00000000021 |
| SPAAR | 0.29 | 2.12 | 0.89 | 2.87 | 0.02 | 1.62 | 0.000000006 |
| COCH | 0.43 | 1.19 | 1.25 | 1.47 | 0.0000697 | 1.54 | 0.000192 |
| MEGF6 | 0.26 | 2.39 | 0.95 | 3.20 | 0.00000000002 | 1.87 | 2,00E-52 |
| LCTL | 1.74 | 3.95 | 3.48 | 1.18 | 0.000297 | 1 | 0.00000009 |
| EPAS1* | 3.56 | 7.40 | 7.92 | 1.06 | 1.5E-32 | 1.15 | 3.1E-27 |
| ETS2 | 3.11 | 6.68 | 7.56 | 1.10 | 1.8E-27 | 1.28 | 6.6E-19 |
| RNF152 | 0.10 | 0.40 | 0.27 | 2 | 0.00169 | 1.43 | 0.00119 |
| FGF9* | 0 | 0.11 | 0.11 | 3.46 | 0.01 | 3.46 | 0.01 |
| FGFR2* | 0.12 | 0.64 | 0.34 | 2.42 | 0.01 | 1.50 | 0.00000148 |

| | | | | | | | |
|---|---|---|---|---|---|---|---|
| FGFR4* | 0.12 | 0.39 | 0.48 | 1.70 | 0.00243 | 2 | 0.02 |
| ITGA11*# | 8.71 | 33.83 | 18.63 | 1.96 | 4.7E-75 | 1.10 | 0,00E+00 |
| LIMCH1 | 0.19 | 1.27 | 0.67 | 2.74 | 0.0000005 | 1.82 | 8.2E-21 |
| PDZD2* | 0.12 | 0.24 | 0.26 | 1 | 0.00206 | 1.12 | 0.02 |
| FLG | 0.08 | 0.27 | 0.50 | 1.75 | 0.000000000000000037 | 2.64 | 0.0000124 |
| FRMD4B | 0.58 | 1.47 | 1.76 | 1.34 | 0.000000000092 | 1.60 | 0.0000002 |
| MTUS2 | 0.25 | 0.94 | 0.52 | 1.91 | 0.03 | 1.06 | 0.0000007 |
| FMOD | 4.01 | 15.95 | 8.61 | 1.99 | 1.3E-20 | 1.10 | 1.5E-92 |
| PIK3R5 | 0.04 | 0.19 | 0.24 | 2.25 | 0.0042 | 2.58 | 0.03 |
| ARHGAP30 | 0.02 | 0.16 | 0.14 | 3 | 0.03 | 2.81 | 0.02 |
| FAM19A5 | 0.02 | 0.31 | 0.24 | 3.95 | 0.02 | 3.58 | 0.00329 |
| GIMAP2 | 0.04 | 1.06 | 1.32 | 4.73 | 0.0000001 | 5.04 | 0.00000529 |
| TIAM2 | 0.53 | 1.20 | 1.93 | 1.18 | 8.2E-19 | 1.86 | 0.00000171 |
| GREM1*# | 60.46 | 241.70 | 183.35 | 2.00 | 0 | 1.60 | 0,00E+00 |
| GDNF | 2.30 | 5.81 | 5.14 | 1.34 | 4.1E-23 | 1.16 | 3.6E-27 |
| GJB2 | 0.31 | 1.23 | 0.97 | 1.99 | 0.00123 | 1.65 | 0.0000168 |
| GNAL | 0.22 | 0.59 | 0.45 | 1.42 | 0.02 | 1.03 | 0.000107 |
| NHLRC4 | 0.18 | 0.57 | 0.52 | 1.66 | 0.05 | 1.53 | 0.03 |
| CDRT4 | 0 | 0.51 | 0.39 | 5.67 | 0.000122 | 5.29 | 0.00000579 |
| WDR62 | 0.46 | 0.93 | 1.27 | 1.02 | 0.000000004 | 1.47 | 0.00202 |
| TRIB2 | 2.34 | 6.39 | 5.42 | 1.45 | 1,00E-20 | 1.21 | 9.9E-33 |
| GUCA1B | 0 | 0.21 | 0.19 | 0.02 | 4.39 | 0.02 | 4.25 |
| HLA-DPA1 | 1.23 | 3.84 | 2.73 | 1.64 | 0.000000002 | 1.15 | 0.000271 |
| HLA-DPB1 | 0.36 | 0.76 | 1.35 | 1.08 | 0.00739 | 1.91 | 0.000000002 |
| IGSF3 | 0.15 | 0.35 | 0.60 | 1.22 | 0.00736 | 2 | 0.00000006 |
| TSPAN33 | 0.03 | 0.22 | 0.27 | 2.87 | 0.03 | 3.17 | 0.05 |
| FMN1 | 0.24 | 0.98 | 1.89 | 2.03 | 3.5E-21 | 2.98 | 1.4E-59 |
| RBPMS2 | 0.43 | 1.21 | 1.55 | 1.49 | 0.00192 | 1.85 | 0.0000212 |
| IL2RB | 0.15 | 0.37 | 0.58 | 1.30 | 0.03 | 1.95 | 0.000117 |
| ITGB8* | 0.08 | 0.19 | 0.20 | 1.25 | 0.04 | 1.32 | 0.01 |
| KCNJ15 | 1.82 | 5.05 | 4.50 | 1.47 | 6,00E-24 | 1.31 | 0.0000000000000000029 |
| FAM174B | 0.50 | 1.12 | 1.45 | 1.16 | 0.00647 | 1.54 | 0.0000384 |
| LY75 | 0 | 0.06 | 0.18 | 2.58 | 0.03 | 4.17 | 0.0000043 |
| MARK1 | 0.31 | 0.69 | 0.62 | 1.15 | 0.02 | 1 | 0.02 |
| MME# | 31.88 | 70.20 | 86.26 | 1.14 | 5.25E-302 | 1.44 | 0,00E+00 |
| MMP1# | 0.13 | 31.71 | 63.18 | 7.93 | 0 | 8.92 | 0,00E+00 |
| MMP9*# | 4.11 | 21.62 | 36.24 | 2.40 | 2.33E-111 | 3.14 | 3.84E-242 |
| MT1A | 2.80 | 7.45 | 6.91 | 1.41 | 0.00493 | 1.30 | 0.02 |
| TNFRSF11B* | 0.13 | 0.90 | 0.97 | 2.79 | 0.00000609 | 2.90 | 0.00000168 |
| STMN3# | 7.27 | 14.89 | 16.83 | 1.03 | 1.5E-24 | 1.21 | 5,00E-34 |
| ADA2# | 0.85 | 5.86 | 4.28 | 2.79 | 8.2E-59 | 2.33 | 5.5E-34 |
| PGM5* | 0.27 | 1.04 | 0.91 | 1.95 | 0.00000177 | 1.75 | 0.0000567 |
| SERPINA1 | 0.45 | 1.01 | 1.52 | 1.17 | 0.00269 | 1.76 | 0.0000007 |
| SPA17 | 2.73 | 6.61 | 7.05 | 1.28 | 0.00000758 | 1.37 | 0.00000127 |
| CLIC6 | 0.47 | 2.32 | 1.55 | 2.30 | 2,00E-20 | 1.72 | 0.000000003 |
| SPET4 | 0.52 | 2.35 | 1.75 | 2.18 | 0.0000003 | 1.75 | 0.00000001 |
| NMRK1 | 2.60 | 6.95 | 6.02 | 1.42 | 0.00000000000036 | 1.21 | 0.000000000000005 |
| NLRP2 | 0.05 | 0.39 | 0.40 | 2.96 | 0.00025 | 3 | 0.000171 |
| GPRC5C | 0.56 | 2.46 | 1.40 | 2.14 | 0.00000000019 | 1.32 | 0.00542 |
| PCDHB3* | 0.05 | 0.30 | 0.24 | 2.58 | 0.00324 | 2.26 | 0.02 |
| PCDHB2* | 0.28 | 1.37 | 0.86 | 2.29 | 0.00000003 | 1.62 | 0.00107 |
| ASPHD2 | 0.40 | 0.89 | 1.11 | 1.15 | 0.00479 | 1.47 | 0.000083 |
| VANGL2 | 0.05 | 0.61 | 0.20 | 3.61 | 0.000000000089 | 2 | 0.00684 |
| TENM2* | 0.23 | 6.69 | 7.17 | 4.86 | 1.42E-268 | 4.96 | 2.36E-282 |
| PCDH19* | 0.09 | 0.19 | 0.19 | 1.08 | 0.04 | 1.08 | 0.05 |

| | | | | | | | |
|---|---|---|---|---|---|---|---|
| CADM3* | 0.09 | 2.76 | 0.30 | 4.94 | 2.4E-38 | 1.74 | 0.00684 |
| PTPRB* | 0.24 | 0.67 | 0.48 | 1.48 | 0.0000106 | 1 | 0.00729 |
| RARRES2# | 7.05 | 62.86 | 52.14 | 3.16 | 5.96E-120 | 2.89 | 6.1E-87 |
| MRPL23# | 9.72 | 36.92 | 40.75 | 1.93 | 1.5E-40 | 2.07 | 1.1E-47 |
| CCL11* | 1.32 | 6.88 | 3.42 | 2.38 | 0.00000000000047 | 1.37 | 0.00148 |
| GPSM3 | 1.46 | 3.61 | 3.14 | 1.31 | 0.000013 | 1.10 | 0.000605 |
| SEMA3F | 0.18 | 1.23 | 0.42 | 2.77 | 0.00000161 | 1.22 | 0.05 |
| GREM2 | 1.30 | 5.10 | 5.32 | 1.97 | 9.4E-38 | 2.03 | 1.1E-39 |
| CBWD6 | 1.77 | 3.77 | 4.60 | 1.09 | 0.0000446 | 1.38 | 0.0000707 |
| LOC644634 | 0.07 | 0.43 | 0.28 | 2.62 | 0.000147 | 2 | 0.01 |
| SLC1A4 | 4.03 | 12.27 | 12.24 | 1.61 | 3.5E-73 | 1.60 | 2.7E-72 |
| WNK3 | 0.01 | 0.07 | 0.06 | 2.81 | 0.02 | 2.58 | 0.02 |
| ACTR3C | 0.17 | 0.56 | 0.96 | 1.72 | 0.00353 | 2.50 | 0.00000001 |
| LRRC61 | 0.13 | 2.16 | 3.47 | 4.05 | 0.000000000000000 93 | 4.74 | 4.3E-27 |
| STAT4 | 0.56 | 1.48 | 1.37 | 1.40 | 0.0000647 | 1.29 | 0.000432 |
| SULT1A1 | 0.44 | 1.56 | 1.49 | 1.83 | 0.00994 | 1.76 | 0.00183 |
| TBX1* | 0.35 | 2.82 | 4.08 | 3.01 | 0.000000000000000 22 | 3.54 | 7.1E-27 |
| TCF21 | 0.10 | 0.83 | 0.63 | 3.05 | 0.000508 | 2.66 | 0.000954 |
| TLE2 | 1.51 | 4.27 | 3.27 | 1.50 | 0.000000000000048 | 1.11 | 0.0000005 |
| TNNT1 | 1.23 | 3.32 | 2.61 | 1.43 | 0.000131 | 1.09 | 0.02 |
| TRPC3 | 0.01 | 0.33 | 0.15 | 5.04 | 0.0000575 | 3.91 | 0.02 |
| TRPM2 | 0 | 0.14 | 0.09 | 3.81 | 0.03 | 3.17 | 0.04 |
| RGPD8 | 1.07 | 3.91 | 5.28 | 1.87 | 3.8E-47 | 2.30 | 1.8E-82 |
| NUTM2D | 0.12 | 0.35 | 0.29 | 1.54 | 0.00551 | 1.27 | 0.03 |
| ALDH5A1 | 0.11 | 0.30 | 0.27 | 1.45 | 0.03 | 1.30 | 0.05 |
| FSD1 | 0.77 | 2.28 | 1.67 | 1.57 | 0.0000117 | 1.12 | 0.00614 |
| CCNJL | 0.11 | 0.35 | 0.41 | 1.67 | 0.04 | 1.90 | 0.00924 |
| CLMN | 0.16 | 1.01 | 0.72 | 2.66 | 0.00000000018 | 2.17 | 0.00000932 |
| SVEP1* | 0.74 | 2.51 | 4.46 | 1.76 | 4.4E-48 | 2.59 | 2.66E-135 |
| MYO15B | 0.10 | 0.26 | 0.28 | 1.38 | 0.02 | 1.49 | 0.02 |
| PDCD1LG2 | 0.22 | 0.70 | 1.14 | 1.67 | 0.00674 | 2.37 | 0.00000247 |
| SLC19A3 | 0.02 | 0.16 | 0.20 | 3 | 0.02 | 3.32 | 0.00533 |
| ITIH5 | 0.24 | 0.82 | 0.51 | 1.77 | 0.00000000047 | 1.09 | 0.00105 |
| LIMD2 | 2.66 | 9.84 | 6.94 | 1.89 | 1.3E-20 | 1.38 | 0.00000004 |
| AP3B2 | 0.10 | 0.57 | 0.50 | 2.51 | 0.0000124 | 2.32 | 0.000135 |
| LRRC3 | 0.35 | 0.87 | 0.71 | 1.31 | 0.0000114 | 1.02 | 0.00166 |
| TCF7L1 | 1.67 | 3.46 | 3.37 | 1.05 | 0.0000006 | 1.01 | 0.00000189 |
| CCDC3 | 0.26 | 0.88 | 0.59 | 1.76 | 0.00022 | 1.18 | 0.03 |
| FAM167A | 0.21 | 1.08 | 0.72 | 2.36 | 0.0000000002 | 1.78 | 0.0000329 |
| ATG10 | 1.37 | 3.53 | 3.02 | 1.37 | 0.000837 | 1.14 | 0.00785 |
| SYT15 | 0.37 | 0.90 | 0.89 | 1.28 | 0.0000139 | 1.27 | 0.000308 |
| OBSCN | 0.05 | 0.12 | 0.10 | 1.26 | 0.000095 | 1 | 0.00513 |
| ACSS1 | 0.60 | 1.40 | 1.94 | 1.22 | 0.0000429 | 1.69 | 0.000000000035 |
| ST6GAL2 | 0.09 | 0.23 | 0.22 | 1.35 | 0.00349 | 1.29 | 0.00349 |
| FNDC1 | 0.56 | 8.02 | 4.42 | 3.84 | 4.17E-182 | 2.98 | 4,00E-78 |
| PYROXD2 | 2.12 | 5.93 | 5.16 | 1.48 | 0.000000000000003 | 1.28 | 0.000000000001 |
| GAS7 | 3.31 | 7.73 | 8.12 | 1.22 | 5.1E-56 | 1.29 | 3.6E-62 |
| DISP2 | 0.16 | 0.74 | 0.68 | 2.21 | 0.00000007 | 2.09 | 0.0000008 |
| ZNF439 | 0.16 | 0.49 | 0.57 | 1.61 | 0.03 | 1.83 | 0.00821 |
| RASL10B | 0.12 | 0.34 | 0.44 | 1.50 | 0.03 | 1.87 | 0.00383 |
| DEPDC7 | 1.70 | 3.73 | 4.96 | 1.13 | 0.0000168 | 1.54 | 0.00000000011 |
| HELB | 0.51 | 1.06 | 1.02 | 1.06 | 0.00195 | 1 | 0.000605 |
| SLIT2# | 10.73 | 21.61 | 32.82 | 1.01 | 4.94E-107 | 1.61 | 0,00E+00 |
| LENG9 | 0.91 | 1.85 | 1.88 | 1.02 | 0.00627 | 1.05 | 0.00449 |

| | | | | | | | |
|---|---|---|---|---|---|---|---|
| GNG8 | 0.18 | 0.44 | 0.71 | 1.29 | 0.03 | 1.98 | 0.000129 |
| RPH3AL | 0.23 | 0.87 | 1.04 | 1.92 | 0.000242 | 2.18 | 0.00000674 |
| CD40 | 1.36 | 3.41 | 4.34 | 1.33 | 0.0000114 | 1.67 | 0.000000002 |
| CELSR1* | 0.16 | 1.10 | 0.39 | 2.78 | 3,00E-34 | 1.29 | 0.000701 |
| TRIM14 | 0.89 | 2.66 | 3.98 | 1.58 | 0.000000000000000 38 | 2.16 | 2.9E-35 |
| NUAK1*# | 6.82 | 27.15 | 14.97 | 1.99 | 0 | 1.13 | 1.7E-78 |
| ARNT2 | 0.59 | 1.66 | 1.22 | 1.49 | 0.00000000000015 | 1.05 | 0.00000601 |
| CDC25C | 0.85 | 1.78 | 3.81 | 1.07 | 0.00611 | 2.16 | 0.000000000000032 |

ASC, adipose-derived stem cell; FPKM, fragments per kilobase of exon model per million reads mapped; FDR, the false discovery rate; *, 31 significantly upregulated genes involved in wound healing-related processes based on Figure 5B; #, 14 most significantly upregulated genes based on Figure 5C.
